# Mendelian randomization study of smoking exposure in relation to primary ovarian failure

**DOI:** 10.1097/MD.0000000000050289

**Published:** 2026-08-21

**Authors:** Danial Habibi, Majid Zaki-Dizaji, Reza Saeedinia, Ava Hemmat, Zohreh Heidary

**Affiliations:** a Social Determinants of Health Research Center, Health Research Institute, Babol University of Medical Sciences, Babol, I.R.Iran; b Legal Medicine Research Center, Legal Medicine Organization, Tehran, Iran; c Student Research Committee, School of Medicine, IRAN University of Medical Sciences, Tehran, Iran; d Department of Nutrition, Faculty of Medicine, Mashhad University of Medical Sciences, Mashhad, Iran; e Vali-E-Asr Reproductive Health Research Center, Family Health Research Institute, Tehran University of Medical Sciences, Tehran, Iran.

**Keywords:** Association, Mendelian randomization, Primary ovarian failure, Smoking

## Abstract

Although observational data suggest a relationship between primary ovarian insufficiency (POI) and smoking, there remain questions about causality. An unhealthy lifestyle, including smoking, can accelerate the depletion of the follicular pool and the early onset of menopause. Extensive research has been conducted on the detrimental effects of tobacco smoke on the ovaries. Mendelian randomization (MR) is a method that uses genetic variants as instruments for making causal inferences about an exposure and an outcome. SNPs associated with smoking behaviors were assessed from recent work by the GWAS and Sequencing Consortium of Alcohol and Nicotine Use (GSCAN), which analyzed up to 1.2 million participants. For the exposure variable, genetic variants associated with smoking behavior were selected, specifically focusing on the quantitative trait cigarettes per day (CPD)—the average number of cigarettes smoked daily by ever-smokers. We then applied GWAS summary statistics from FinnGen consortium data for POI. The dataset included 254 POF cases and 118,228 controls. We identified 22 SNPs associated with CPD to serve as instrumental variables. Genetic predisposition to higher cigarettes per day showed a suggestive but nonsignificant association with increased POI risk (OR = 1.443, 95% CI: 0.731–2.849, *P* = .289). We found limited evidence for an association between genetically predicted cigarettes per day and POI and indicate that moderate effects cannot be ruled out. There was little evidence of heterogeneity [Q_IVW_ = 21.19, *P* = .447; Q_Egger_ = 21.16, *P* = .387; I^2^ = 5.5%, 95% CI: 0.0%, 38.2%; I^2^_GX_ = 97.24%] and pleiotropy [MR-Egger_intercept_ = 0.0063, *P* = .872; MR-PRESSO_Global Test_ = 0.516]. This finding was consistently supported across multiple analytical methods. We found no strong evidence of a causal association between genetically predicted cigarettes per day and POI under the current study design; however, moderate effects cannot be excluded.

## 1. Introduction

Primary ovarian insufficiency/failure (POI/POF) is a spectrum disorder that not only impacts female fertility but also contributes to morbidity and mortality associated with the long-term deficiency of estrogen. POI typically manifests in women under 40 years of age and is diagnosed in the presence of amenorrhea lasting 4–6 months, along with elevated follicle-stimulating hormone (FSH) levels and decreased estradiol levels confirmed in 2 tests conducted 1 month apart. Although smoking has been consistently associated with earlier natural menopause, it is important to distinguish physiological ovarian aging from pathological premature ovarian insufficiency. While smoking appears to accelerate the timing of natural menopause, whether it directly causes the distinct pathological entity of POI remains unclear.^[1]^ POI is hypothesized to stem from either follicular dysfunction or follicular depletion, although the exact mechanisms driving its development remain elusive. Remarkably, about 90% of spontaneous POI cases lack a clear underlying cause.^[1]^ Environmental factors such as smoking, nicotine, and related substances like dimethylbenzanthracene are thought to contribute to POI by binding to receptors on ovarian granulosa cells. This interaction triggers proapoptotic gene activation and inhibits aromatase activity, ultimately reducing circulating estradiol levels.^[1]^ Smoking, in particular, is well-documented for its detrimental effects on female fertility and is a recognized risk factor for POI.^[2]^ Notably, genetic and hormonal factors tied to the menstrual cycle may make smoking initiation and cessation more sensitive to sex-specific influences in women than in men, suggesting that tobacco-dependence interventions could benefit from accounting for this difference.^[3]^

While smoking is known to accelerate the timing of physiological menopause, this does not necessarily imply a causal role in pathological premature ovarian failure, which is biologically and etiologically distinct. Evidence from large prospective cohorts and genetic studies indicates that smoking is associated with earlier menopause and accelerated ovarian aging,^[4,5]^ although its role in POI remains uncertain. Components of tobacco smoke have been shown to interfere with follicle formation through several converging mechanisms—triggering programmed cell death and autophagic activity, causing genomic damage, and disrupting the normal signaling between oocytes and their surrounding granulosa cells—ultimately contributing to loss of ovarian follicles.^[6]^ Cigarette smoke exposure has repeatedly been linked to a reduced ovarian reserve,^[7]^ and in some cases, to an earlier-than-expected menopause.^[5]^ Notably, evidence suggests that maternal smoking during pregnancy is associated with fewer oocytes and somatic cells in the ovaries of female offspring,^[8]^ which may translate into lower fertility and an earlier menopausal transition later in life.^[9]^ Supporting evidence comes from a murine model of cigarette-smoke-induced chronic obstructive pulmonary disease, in which nasal smoke exposure caused female mice to lose primordial follicles, undergo apoptosis within antral (sinus) follicles, show heightened oxidative stress, and ovulate less often.^[10]^ More broadly, smoke exposure has also been tied to a shrinking follicle pool, altered estrus cyclicity, and changes in circulating hormone levels.^[6]^ Together, these findings point to a substantial and potentially multigenerational impact of smoking on ovarian health. Heavier smoking in particular has been linked to earlier menopause,^[11,12]^ with smokers reaching menopause roughly a year sooner than nonsmokers on average.^[5]^ A genetic analysis further estimated that age at natural menopause (ANM) declined by roughly 2.5 weeks for each additional cigarette smoked per day, consistent with a dose-dependent, and at least partly heritable, relationship linking tobacco and alcohol use to earlier menopause.^[4]^ Cessation appears to partially offset this risk, as earlier quitting is associated with a lower likelihood of early menopause. Evidence on whether secondhand exposure carries the same risk as active smoking is mixed: some studies implicate both passive and active smoking,^[13]^ while others find an effect only among active smokers.^[6]^ Whether tobacco use, both in terms of quantity and duration, is associated with earlier onset of natural menopause or POI remains to be determined.

Observational associations between smoking and primary ovarian insufficiency are particularly vulnerable to confounding, as several determinants of smoking behavior are also independently associated with reproductive health. For example, lower socioeconomic status is linked to higher smoking prevalence, increased psychosocial stress, poorer nutrition, and reduced access to preventive and specialist reproductive healthcare, all of which may influence the likelihood of POI diagnosis. Similarly, smoking frequently clusters with other health-related behaviors, including alcohol consumption, higher or lower body mass index, and comorbid conditions, making it difficult to disentangle the independent effect of smoking from correlated lifestyle and healthcare factors in conventional observational analyses. We employed Mendelian randomization to investigate the causal impact of individual smoking behavior, quantified as cigarettes per day, on the development of primary ovarian insufficiency.

## 2. Method

### 2.1. Study design and data sources

**Figure 1.**
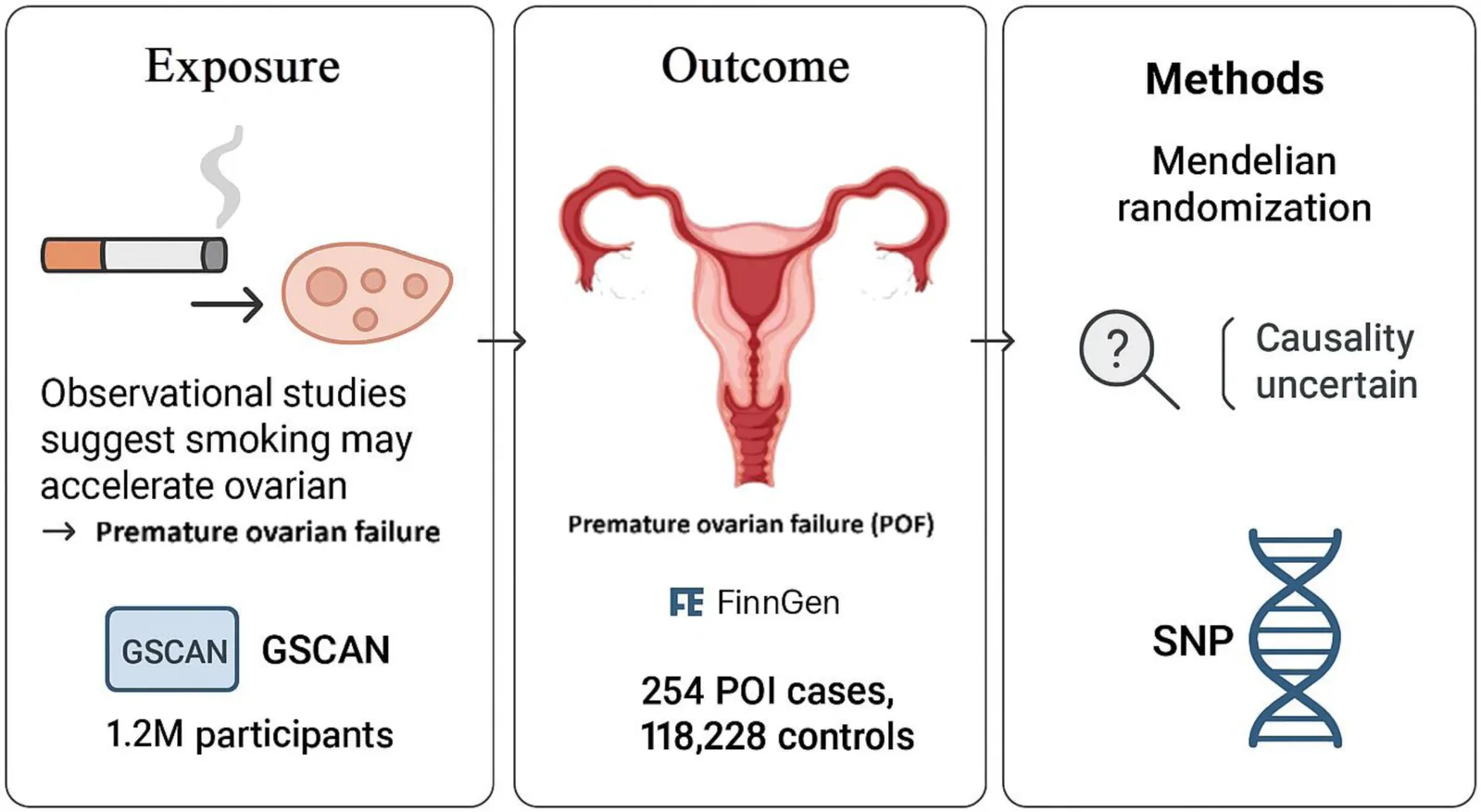
Two-sample Mendelian randomization study of smoking exposure in relation to primary ovarian failure. Exposure is the average number of cigarettes smoked daily by ever-smokers. The outcome is primary ovarian failure.

This study utilized a two-sample Mendelian randomization (TSMR) design, relying on GWAS summary-level data, to explore the potential causal link between smoking (as the exposure) and primary ovarian failure (POF) (as the outcome) in individuals of European ancestry (Fig. 1). The analysis adhered to the STROBE-MR reporting guidelines.^[14]^

For the exposure variable, genetic variants associated with smoking behavior were selected, specifically focusing on the quantitative trait cigarettes per day (CPD)—the average number of cigarettes smoked daily by ever-smokers. These data were drawn from the GWAS and Sequencing Consortium of Alcohol and Nicotine Use (GSCAN), which analyzed up to 1.2 million participants.^[15]^ That study identified 566 genetic variants across 406 loci linked to various stages of tobacco use (initiation, cessation, and intensity) and alcohol consumption (drinks per week). Of these, 55 SNPs were genome-wide significant and conditionally independent, accounting for 1.09% of the variance in CPD among 337,334 European-ancestry ever-smokers. CPD was selected as the primary exposure because it provides a continuous measure of smoking intensity with strong, well-replicated genetic instruments from large-scale GWAS. Compared to binary traits such as smoking initiation, CPD allows for more precise estimation of dose–response relationships. However, CPD does not capture duration, cumulative exposure, or timing of smoking behavior, which may be biologically relevant for ovarian aging and POI.

Data on the outcome, primary ovarian failure, were sourced from the FinnGen (finn-b-E4_OVARFAIL) project (https://gwas.mrcieu.ac.uk/datasets/finn-b-E4_OVARFAIL/), which includes European-ancestry participants.^[16]^ The dataset included 254 POF cases and 118,228 controls, covering 16,379,677 SNPs. POF was defined using ICD-10 codes to reflect estrogen deficiency, premature menopause, and resistant ovarian syndrome, explicitly excluding cases related to menopause, female climacteric states, simple gonadal dysgenesis, and Turner syndrome. GSCAN and FinnGen are independent cohorts with no known/meaningful sample overlap.

### 2.2. Selection of SNPs associated with smoking exposure

In our study, we implemented comprehensive quality control procedures to ensure the reliability of selected SNPs. We began by applying a strict genome-wide significance threshold of *P* < 5 × 10^−8^ for identifying valid instrumental variables. To eliminate correlated variants and retain only independent genetic instruments, we conducted linkage disequilibrium (LD) clumping using the TwoSampleMR package’s default parameters (R^2^ = 0.001 and a clumping window of 10,000 kb) and the European ancestry reference panel from the 1000 Genomes Project (Phase 3). Within each LD block, only the SNP with the lowest *P*-value was retained. In cases where a target SNP was unavailable in the outcome dataset, proxy variants in high linkage disequilibrium (R^2^ ≥ 0.8) were used based on the European 1000 Genomes reference panel.^[17]^

Instrument strength was evaluated using the F-statistic (F = β^2^/SE^2^), and we excluded all SNPs with F-values below 10 to ensure robust instrument validity.^[18]^ To strengthen robustness, we conducted an additional sensitivity analysis excluding SNPs with F-statistics < 30. Additional filtering removed SNPs with a minor allele frequency (MAF) <0.01.^[19]^ During harmonization, we excluded ambiguous palindromic SNPs with intermediate allele frequencies (between 0.42 and 0.58) to avoid strand alignment errors.^[20]^ Finally, Steiger filtering was applied to assess directionality; no SNPs were excluded, and all instruments showed stronger associations with the exposure than with the outcome.^[21]^

### 2.3. Mendelian randomization analysis

For the univariate Mendelian randomization analysis, we primarily used the inverse variance weighted (IVW) method within a random-effects framework.^[22]^ Given the potential for pleiotropy or the inclusion of invalid instruments to bias IVW estimates, we applied a range of additional methods to strengthen causal inference. These included MR-Egger regression, the weighted median estimator, and both simple and weighted mode-based approaches.^[23]^ To further reinforce the robustness of our results, we incorporated advanced methods such as maximum likelihood estimation, Robust Adjusted Profile Score (RAPS), MR-Lasso, constrained maximum likelihood, mode-based estimation, debiased IVW, and the contamination mixture model.^[24–26]^ This diverse analytical strategy was designed to comprehensively test the consistency and credibility of the observed causal relationships.

We also carried out extensive sensitivity analyses to assess the reliability of our findings. Heterogeneity among genetic instruments was evaluated using Cochran’s Q statistic and I^2^ metrics for IVW, as well as Rücker’s Q test in the MR-Egger framework, with *p*-values above .05 suggesting low heterogeneity.^[27]^ To detect horizontal pleiotropy, we used both the MR-Egger intercept and the MR-PRESSO global test,^[28]^ while potential outlier SNPs were identified using MR-PRESSO and radial MR methods.^[29,30]^ Influential genetic variants were further examined using Cook’s distance and Studentized residuals, and effect estimates were recalculated after excluding any outliers. Leave-one-out analyses were performed to determine if any single SNP disproportionately influenced the overall result,^[31]^ and funnel plots were visually inspected to detect asymmetry indicative of pleiotropy.^[28]^

All analyses were conducted in RStudio, using the “TwoSampleMR” package for core MR procedures, “MendelianRandomization” for extended methods, and “mr.raps” for robust estimation. Visualization and result presentation were supported by the “forestploter,” “meta,” “grid,” and “ggplot2” packages. Additionally, the “mrrobust” package in STATA was used to complement and cross-validate the results.

## 3. Results

### 3.1. Main results

This analysis investigated a potential causal link between smoking and primary ovarian insufficiency using Mendelian randomization. Following established criteria (GWAS significance threshold of *P* < 5 × 10^−8^ and clumping for independence), 23 single nucleotide polymorphisms (SNPs) associated with smoking were selected as instrumental variables. Data harmonization ensured consistency, retaining all 23 SNPs with no palindromic conflicts. The strength of these instruments was confirmed by high F-statistics (range: 29.90–961), and a Steiger directionality test ruled out reverse causation. The genetic instruments explained approximately 1.09% of the variance in cigarettes per day, consistent with the original GSCAN GWAS, and all SNPs had F-statistics > 10, indicating strong instrument strength.

**Figure 2.**
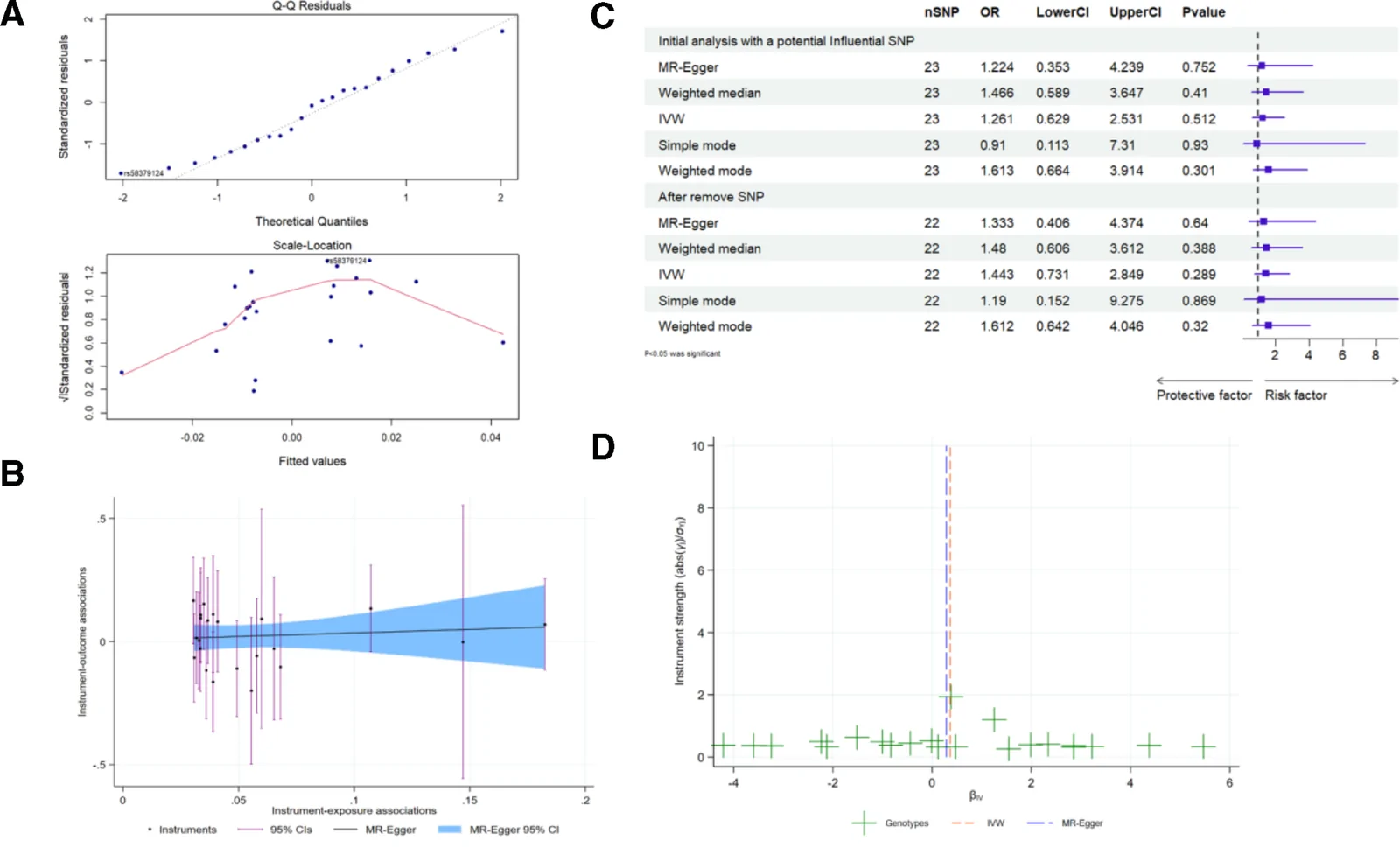
Assesses the potential influence of individual genetic variants (SNPs). Panel A: Identifies the SNP rs58379124 (located near the CHRNB3 gene) as potentially influential. Panel B: Shows that the data points align closely with the regression line, indicating little heterogeneity, and the line’s intercept at zero suggests no directional pleiotropy. Panel C: Compares the results before and after the removal of the influential SNP rs58379124. After excluding the influential SNP, all methods yield odds ratios above one. Panel D: Presents a funnel plot where the symmetrical distribution of points confirms the absence of outlier SNPs.

**Figure 3.**
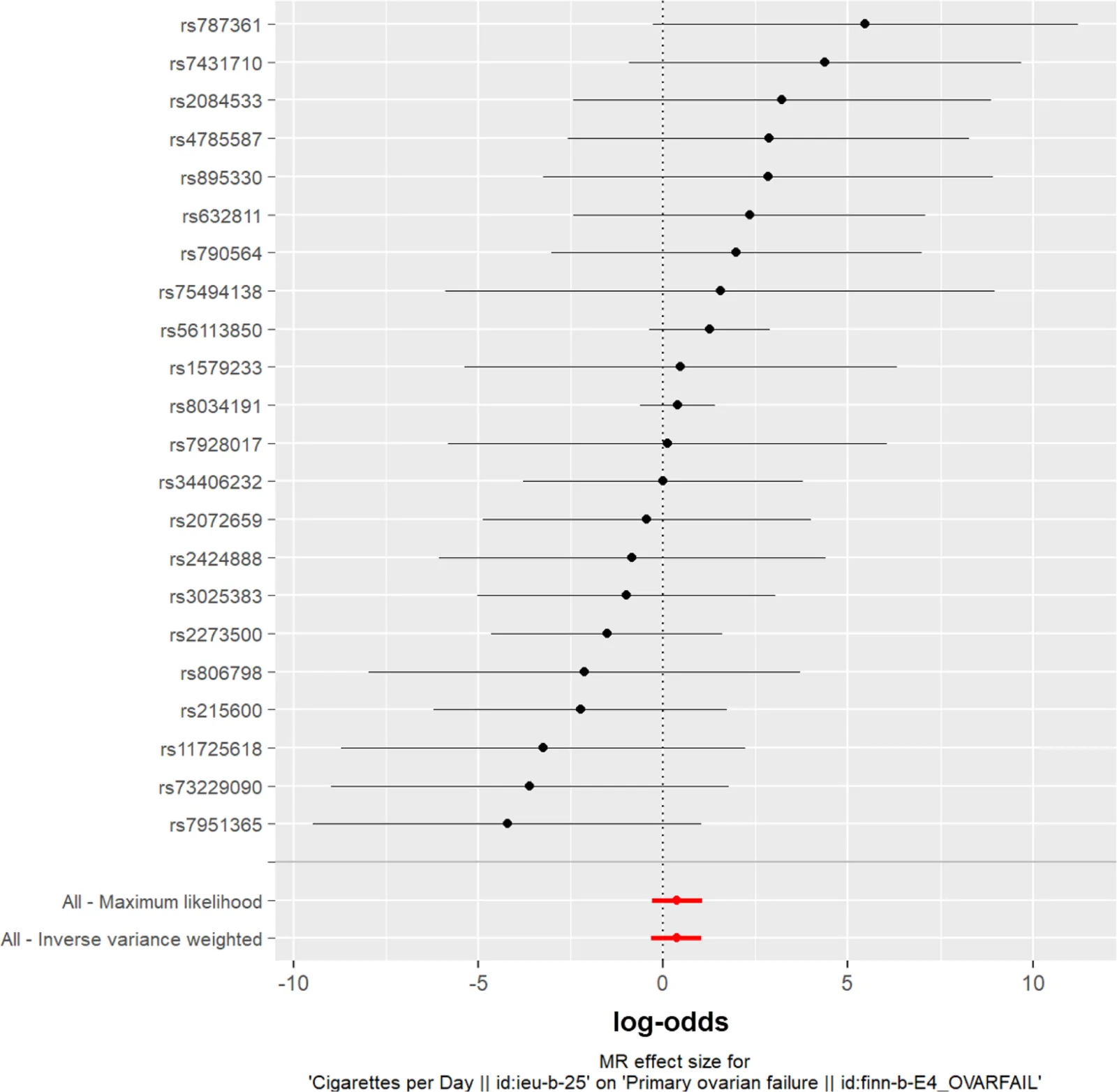
Forest plot. The plot visualizes the causal estimates (β coefficients on the log-odds scale) for each of the 22 instrumental SNPs (rows). The horizontal lines represent the 95% confidence intervals for each SNP-specific estimate.

All 23 SNPs were successfully harmonized. Following the identification of rs58379124 as an influential outlier, this SNP was excluded from the primary analysis, leaving 22 SNPs in the final IVW model. Following the removal of the influential SNP rs58379124 (located near the CHRNB3 gene, as illustrated in Figure 2, and Supplementary data, Supplemental Digital Content 1), the Inverse-Variance Weighted (IVW) analysis found no statistically significant association between smoking and the risk of POI [OR = 1.443, 95%CI: 0.731–2.849, *P* = .289]. There was little evidence of heterogeneity [Q_IVW_ = 21.19, *P* = .447; Q_Egger_ = 21.16, *P* = .387; I^2^ = 5.5%, 95%CI: 0.0%, 38.2%; I^2^_GX_ = 97.24%] and pleiotropy [MR-Egger intercept=0.0063, *P* = .872; MR-PRESSO Global _Test_ = 0.516]. This result was confirmed by several methods, as shown in Figure 3, for example, MR-PRESSO [OR = 1.443, 95%CI: 0.731–2.849, *P* = .301].

We also evaluated several visual sensitivity analyses (Figures S1, Supplemental Digital Content 2). Scatterplots (Figure S2, Supplemental Digital Content 3), leave-one-out (Figure S3, Supplemental Digital Content 4), and funnel plots (Figure S4, Supplemental Digital Content 5) were used to assess the impact of outliers. The association of smoking exposure in relation to primary ovarian failure is depicted through a forest plot (Fig. 3). All genetic variants explained more variance in the exposure than in the outcome, satisfying the MR Steiger directionality criterion; no variants were excluded on this basis. Excluding SNPs with F-statistics < 30 did not materially alter the effect estimates or precision. The direction and magnitude of the association remained similar, with no evidence of increased heterogeneity or pleiotropy (Figure S5, Supplemental Digital Content 6).

## 4. Discussion

The etiology of POI, a multifactorial condition with major health consequences, remains largely unknown. While lifestyle factors such as smoking are known to deplete the ovarian reserve and advance menopause timing, our study of 254 women with POI and a large control group of 118,228 women of European descent found no strong evidence of a causal effect of smoking on POI; however, given the limited statistical power, moderate or clinically meaningful effects cannot be excluded.

Several explanations may account for the null finding, including limited statistical power, an incomplete measure of smoking exposure, etiological heterogeneity within POI, and the restricted ancestry of the study populations; these limitations are discussed in detail below.

Both the exposure (GSCAN) and outcome (FinnGen) datasets are derived from populations of European ancestry. Differences in genetic architecture, smoking behavior, and environmental exposures may limit the generalizability of these findings to other populations. Additionally, POI status in FinnGen is based on registry diagnoses, and any non-differential misclassification of cases would be expected to bias causal estimates toward the null, potentially contributing to the lack of a detected association.

Among environmental risk factors, smoking is well-documented to have detrimental effects on female fertility and is recognized as a contributor to POI.^[3,32]^ More broadly, environmental exposures are recognized as important determinants of ovarian reserve and may influence the timing of menopause, whether encountered prenatally or later in adulthood.^[33]^ Cigarette smoke exposure has been linked to a reduction in ovarian reserve^[34]^ and an increased likelihood of earlier menopause in women who smoke.^[5]^ Meta-analytic evidence also indicates a strong association between cigarette smoking and a higher incidence of dysmenorrhea in women of reproductive age.^[35]^ A 2014 systematic review and meta-analysis from Australia reported that smokers had a 33% greater likelihood than nonsmokers of reaching postmenopause at any given age.^[2]^ Furthermore, maternal smoking during pregnancy has been associated in some studies with a reduction in both oocyte and somatic cell numbers in the ovary,^[8]^ diminished fertility, and earlier menopause in offspring.^[9]^ However, these findings are not entirely consistent, as some studies have reported contradictory results.^[7]^

While our study did not find a causal link between smoking and POI, the relationship between smoking and female reproductive health remains complex and somewhat contradictory based on prior studies. Remarkably, a genetic study by Ruth et al.^[4]^ demonstrated that genetically predicted smoking causally accelerates natural menopause, with each cigarette smoked per day leading to an earlier menopause by around 2.5 weeks, implying a significant effect on ovarian aging. However, other studies using similar MR approaches, such as that by Hernáez et al.,^[36]^ found no causal link between smoking and infertility within the Norwegian MoBa cohort. While Wootton et al.^[37]^ presented mixed results in MoBa, while observational data suggested an association between smoking and longer time to conception, the causal evidence was inconsistent.

Ruth et al. demonstrated that smoking accelerates physiological follicular exhaustion, a gradual depletion advancing menopause by approximately 2.5 weeks per cigarette daily. However, POI represents acute pathological failure often driven by autoimmune attack or genetic defects. Smoking may lack sufficient potency to trigger such pathological mechanisms, even while incrementally depleting the follicular pool. This would explain why MR detects effects on continuous aging phenotypes (menopause timing) but not dichotomous pathological outcomes (POI). These discrepancies can be reconciled by considering several key factors. Smoking may predominantly accelerate physiological ovarian aging and advance the timing of natural menopause without necessarily inducing the pathological ovarian failure that defines POI.^[33]^ Thus, evidence linking smoking to menopause timing should not be assumed to imply causality for POI. Furthermore, the pronounced etiological heterogeneity of conditions like infertility and POI could dilute a causal signal that is only relevant to a specific subset of cases. Finally, methodological differences, including differences in statistical power between these studies and the reliance on “cigarettes per day” as a potentially insufficient proxy for more biologically relevant exposures like initiation or duration of smoking, likely contribute to the inconsistent findings across studies.

The IVW estimate suggested a potential 44% increase in POI odds per genetically predicted increase in CPD, but this did not reach statistical significance, and the confidence interval reflects substantial uncertainty, consistent with limited statistical power.

This study has several limitations that could explain our null findings regarding the effect of smoking (cigarettes per day) on POI. The statistical power to detect modest causal effects was limited. Based on a formal power calculation with 254 cases and 118,228 controls at α = 0.05 and an instrument explaining 1.1% of the variance in cigarettes per day (as reported by GSCAN), the power was below ~30% for an OR of 2.0. Therefore, the null MR findings should be interpreted as “no strong evidence of a large effect,” rather than definitive evidence of no causal effect. Another important limitation of this study is the smoking exposure measure used. CPD among ever-smokers captures smoking intensity but does not reflect duration, cumulative exposure, or timing of smoking behavior. Pack-years, which incorporate both intensity and duration, may be more biologically relevant, as experimental animal models demonstrate dose- and duration-dependent follicular depletion and oxidative damage. Consequently, individuals with short-term high-intensity smoking and long-term lower-intensity smoking may contribute similarly to the CPD instrument despite potentially different biological impacts.

Additionally, Mendelian randomization estimates represent average causal effects across heterogeneous disease mechanisms. POI encompasses autoimmune-mediated ovarian destruction, chromosomal abnormalities, monogenic defects affecting folliculogenesis or meiosis, iatrogenic causes, and idiopathic cases. If smoking primarily influences specific subtypes—such as accelerating age-related decline in women with borderline ovarian reserve—averaging across all POI cases may dilute a subtype-specific signal. Given the limited number of POI cases in the present study (n = 254), stratified analyses by etiological subtype were not feasible, and this heterogeneity may have attenuated detectable associations.

Using genetic instruments for smoking intensity, we found limited evidence for a causal association with POI. However, the point estimate does not exclude a potentially meaningful increase in risk. Limited statistical power, the use of an incomplete exposure proxy (CPD), and the etiological heterogeneity of POI restrict causal interpretation. These findings should therefore be considered inconclusive rather than definitive evidence of no effect.

## Acknowledgments

We want to acknowledge the participants and investigators who made summary data available.

## Author contributions

**Writing – original draft:** Danial Habibi, Zohreh Heidary.

**Writing – review & editing:** Majid Zaki-Dizaji, Reza Saeedinia, Ava Hemmat.













## References

[1] Sopiarz N Sparzak PB. Primary ovarian insufficiency. In: StatPearls [Internet]. StatPearls Publishing; 2026. 36943992

[2] Schoenaker DAJM Jackson CA Rowlands JV Mishra GD. Socioeconomic position, lifestyle factors and age at natural menopause: a systematic review and meta-analyses of studies across six continents. Int J Epidemiol. 2014;43:1542–62. 24771324 10.1093/ije/dyu094 PMC4190515

[3] Cui J Wang Y. Premature ovarian insufficiency: a review on the role of tobacco smoke, its clinical harm, and treatment. J Ovarian Res. 2024;17:8. 38191456 10.1186/s13048-023-01330-y PMC10775475

[4] Ruth KS Day FR Hussain J.; Biobank-based Integrative Omics Study (BIOS) Consortium. Genetic insights into biological mechanisms governing human ovarian ageing. Nature. 2021;596:393–7. 34349265 10.1038/s41586-021-03779-7 PMC7611832

[5] Whitcomb BW Purdue-Smithe AC Szegda KL. Cigarette smoking and risk of early natural menopause. Am J Epidemiol. 2018;187:696–704. 29020262 10.1093/aje/kwx292 PMC5888979

[6] Li F Wang Y Xu M. Single-nucleus RNA Sequencing reveals the mechanism of cigarette smoke exposure on diminished ovarian reserve in mice. Ecotoxicol Environ Saf. 2022;245:114093. 36116238 10.1016/j.ecoenv.2022.114093

[7] Budani MC Tiboni GM. Ovotoxicity of cigarette smoke: a systematic review of the literature. Reprod Toxicol. 2017;72:164–81. 28684319 10.1016/j.reprotox.2017.06.184

[8] Fowler PA Childs AJ Courant F. In utero exposure to cigarette smoke dysregulates human fetal ovarian developmental signalling. Hum Reprod. 2014;29:1471–89. 24847019 10.1093/humrep/deu117

[9] Gold EB Crawford SL Avis NE. Factors related to age at natural menopause: longitudinal analyses from SWAN. Am J Epidemiol. 2013;178:70–83. 23788671 10.1093/aje/kws421 PMC3698989

[10] Sobinoff AP Beckett EL Jarnicki AG. Scrambled and fried: cigarette smoke exposure causes antral follicle destruction and oocyte dysfunction through oxidative stress. Toxicol Appl Pharmacol. 2013;271:156–67. 23693141 10.1016/j.taap.2013.05.009

[11] Hyland A Piazza K Hovey KM. Associations between lifetime tobacco exposure with infertility and age at natural menopause: the Women’s Health Initiative Observational Study. Tob Control. 2016;25:706–14. 26666428 10.1136/tobaccocontrol-2015-052510

[12] Aiken CE Tarry-Adkins JL Penfold NC Dearden L Ozanne SE. Decreased ovarian reserve, dysregulation of mitochondrial biogenesis, and increased lipid peroxidation in female mouse offspring exposed to an obesogenic maternal diet. FASEB J. 2015;30:1548–56. 26700734 10.1096/fj.15-280800 PMC4799509

[13] Ertunc D Tok EC Aytan H Gozukara YM. Passive smoking is associated with lower age at menopause. Climacteric. 2015;18:47–52. 10.3109/13697137.2014.938041 25032840

[14] Sharma J Jangale V Swain AK Yadav P. An optimized instrument variable selection approach to improve causality estimation in association studies. Sci Rep. 2024;14:22781. 39354059 10.1038/s41598-024-73970-z PMC11445377

[15] Liu M Jiang Y Wedow R.; 23andMe Research Team. Association studies of up to 1.2 million individuals yield new insights into the genetic etiology of tobacco and alcohol use. Nat Genet. 2019;51:237–44. 30643251 10.1038/s41588-018-0307-5 PMC6358542

[16] Kurki MI Karjalainen J Palta P.; FinnGen. FinnGen provides genetic insights from a well-phenotyped isolated population. Nature. 2023;613:508–18. 36653562 10.1038/s41586-022-05473-8 PMC9849126

[17] Haycock PC Borges MC Burrows K.; Fatty Acids in Cancer Mendelian Randomization Collaboration. Design and quality control of large-scale two-sample Mendelian randomization studies. Int J Epidemiol. 2023;52:1498–521. 38587501 10.1093/ije/dyad018 PMC10555669

[18] Sanderson E Spiller W Bowden J. Testing and correcting for weak and pleiotropic instruments in two-sample multivariable Mendelian randomization. Stat Med. 2021;40:5434–52. 34338327 10.1002/sim.9133 PMC9479726

[19] Fan B Zhao JV. Genetic proxies for antihypertensive drugs and mental disorders: Mendelian randomization study in European and East Asian populations. BMC Med. 2024;22:6. 38166843 10.1186/s12916-023-03218-6 PMC10763027

[20] Li Y Xie T Vos M Snieder H Hartman CA. Shared genetic architecture and causality between autism spectrum disorder and irritable bowel syndrome, multisite pain, and fatigue. Transl Psychiatry. 2024;14:476. 39580447 10.1038/s41398-024-03184-4 PMC11585586

[21] Hemani G Tilling K Smith GD. Orienting the causal relationship between imprecisely measured traits using GWAS summary data. PLoS Genet. 2017;13:e1007081. 29149188 10.1371/journal.pgen.1007081 PMC5711033

[22] Nikolakopoulou A Mavridis D Salanti G. How to interpret meta-analysis models: fixed effect and random effects meta-analyses. Evid Based Ment Health. 2014;17:64. 24778439 10.1136/eb-2014-101794 PMC13404559

[23] Bowden J Del Greco M F Minelli C Smith GD Sheehan N Thompson J. A framework for the investigation of pleiotropy in two‐sample summary data Mendelian randomization. Stat Med. 2017;36:1783–802. 28114746 10.1002/sim.7221 PMC5434863

[24] Qi G Chatterjee N. Mendelian randomization analysis using mixture models for robust and efficient estimation of causal effects. Nat Commun. 2019;10:1941. 31028273 10.1038/s41467-019-09432-2 PMC6486646

[25] Slob EAW Burgess S. A comparison of robust Mendelian randomization methods using summary data. Genet Epidemiol. 2020;44:313–29. 32249995 10.1002/gepi.22295 PMC7317850

[26] Burgess S Foley CN Allara E Staley JR Howson JMM. A robust and efficient method for Mendelian randomization with hundreds of genetic variants. Nat Commun. 2020;11:1–11. 31953392 10.1038/s41467-019-14156-4 PMC6969055

[27] Hemani G Zheng J Elsworth B. The MR-Base platform supports systematic causal inference across the human phenome. Elife. 2018;7:e34408. 29846171 10.7554/eLife.34408 PMC5976434

[28] Bowden J Smith GD Burgess S. Mendelian randomization with invalid instruments: effect estimation and bias detection through Egger regression. Int J Epidemiol. 2015;44:512–25. 26050253 10.1093/ije/dyv080 PMC4469799

[29] Hemani G Bowden J Smith GD. Evaluating the potential role of pleiotropy in Mendelian randomization studies. Hum Mol Genet. 2018;27:R195–208. 29771313 10.1093/hmg/ddy163 PMC6061876

[30] Verbanck M Chen C-Y Neale B Do R. Detection of widespread horizontal pleiotropy in causal relationships inferred from Mendelian randomization between complex traits and diseases. Nat Genet. 2018;50:693–8. 29686387 10.1038/s41588-018-0099-7 PMC6083837

[31] Burgess S Smith GD Davies NM. Guidelines for performing Mendelian randomization investigations: update for summer 2023. Wellcome open Res. 2023;4:186. 32760811 10.12688/wellcomeopenres.15555.1 PMC7384151

[32] Webber L Davies M Anderson R.; European Society for Human Reproduction and Embryology (ESHRE) Guideline Group on POI. ESHRE Guideline: management of women with premature ovarian insufficiency. Hum Reprod. 2016;31:926–37. 27008889 10.1093/humrep/dew027

[33] Richardson MC Guo M Fauser B Macklon NS. Environmental and developmental origins of ovarian reserve. Hum Reprod Update. 2014;20:353–69. 24287894 10.1093/humupd/dmt057

[34] Gunes S Mahmutoglu AM Arslan MA Henkel R. Smoking‐induced genetic and epigenetic alterations in infertile men. Andrologia. 2018;50:e13124. 30132931 10.1111/and.13124

[35] Qin L-L Hu Z Kaminga AC. Association between cigarette smoking and the risk of dysmenorrhea: a meta-analysis of observational studies. PLoS One. 2020;15:e0231201. 32294123 10.1371/journal.pone.0231201 PMC7159229

[36] Hernáez A Wootton RE Page CM. Smoking and subfertility: multivariable regression and Mendelian randomization analyses in the Norwegian Mother, Father and Child Cohort Study. Fertil Steril. 2022;118:180–90. 35562204 10.1016/j.fertnstert.2022.04.001 PMC7612999

[37] Wootton RE Lawn RB Magnus MC. Associations between health behaviours, fertility and reproductive outcomes: triangulation of evidence in the Norwegian Mother, Father and Child Cohort Study (MoBa). BMC Med. 2023;21:125. 37013617 10.1186/s12916-023-02831-9 PMC10071662

