# Supplementary material for: Mendelian randomization study of smoking exposure in relation to primary ovarian failure
Source: Medicine (Baltimore). 2026 Aug 21;105(34):e50289. doi: 10.1097/MD.0000000000050289 (PMC13502784; doi:10.1097/MD.0000000000050289)

S4: Forest plot

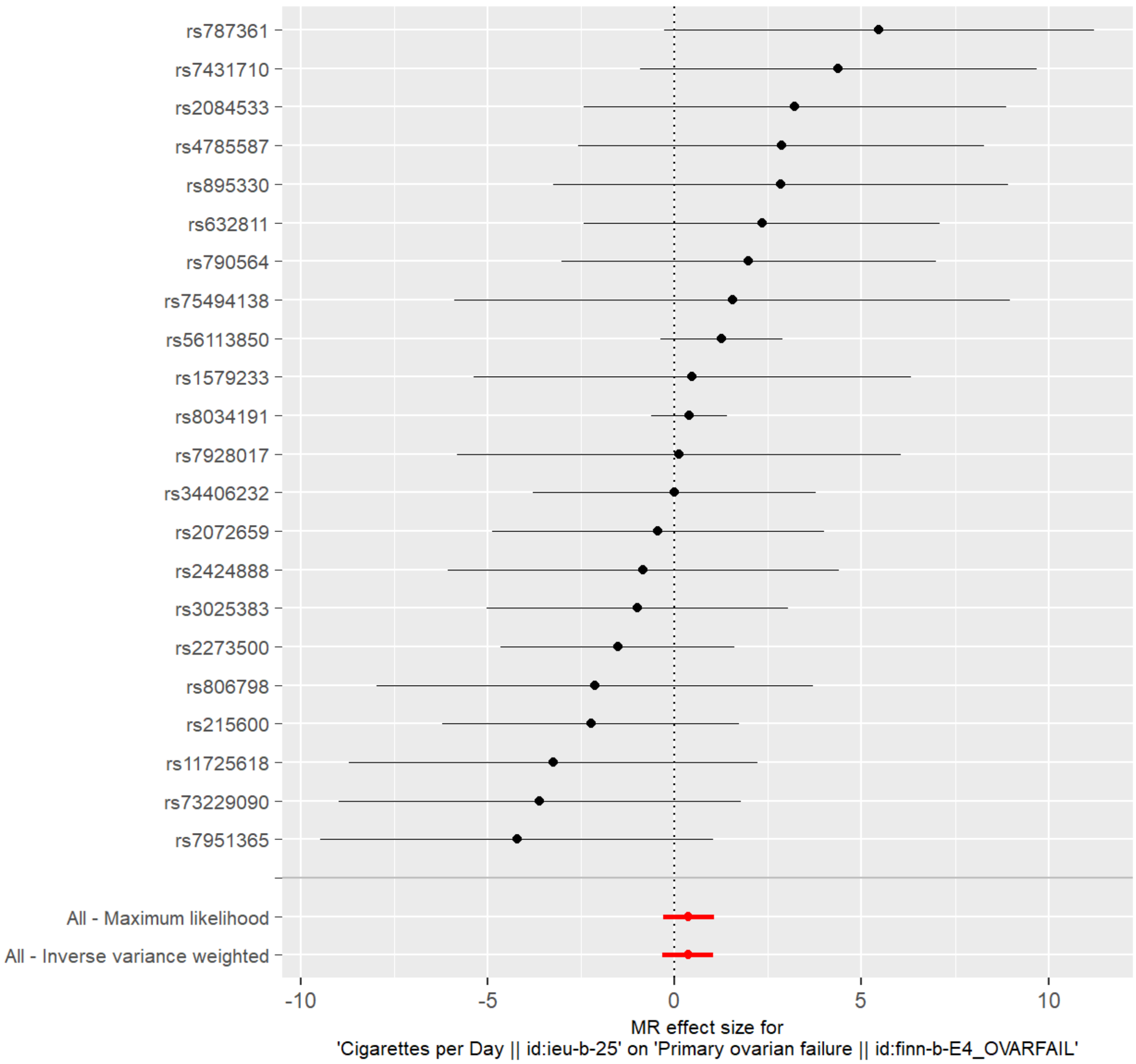

Supplement: Supplementary file 4 [file medi-105-e50289-s005.pdf]
