# Supplementary material for: Mendelian randomization study of smoking exposure in relation to primary ovarian failure
Source: Medicine (Baltimore). 2026 Aug 21;105(34):e50289. doi: 10.1097/MD.0000000000050289 (PMC13502784; doi:10.1097/MD.0000000000050289)
Supplement: Supplementary file 5 [file medi-105-e50289-s006.pdf]

S5: The result without the weak instrument (F<30).

In the inverse-variance weighted (IVW) analysis under a random-effects model including 21 independent variants, no evidence of heterogeneity was observed. Cochran's Q statistic was 18.13 with 20 degrees of freedom (P = 0.579), corresponding to an I^2 of 0.0%, indicating that the SNP-specific causal estimates were statistically consistent with a common underlying effect. The F-statistic was 103.2, substantially exceeding the conventional threshold of 10, suggesting that weak instrument bias is unlikely. Moreover, given the absence of heterogeneity and the non-significant MR-Egger intercept observed in sensitivity analyses, there was no evidence of directional horizontal pleiotropy (intercept= -0.012, P= 0.757). Despite the methodological robustness of the instrument set, the IVW estimate did not demonstrate a statistically significant causal association (OR = 1.34, 95% CI: 0.68–2.66, P = 0.395).

**Method**	**OR**	**95% CI (OR)**	**P-value**
Simple median	1.13	0.31 – 4.08	0.853
Weighted median	1.48	0.58 – 3.74	0.410
Penalized weighted median	1.48	0.58 – 3.74	0.410
IVW	1.34	0.68 – 2.66	0.395
Penalized IVW	1.34	0.68 – 2.66	0.395
Robust IVW	1.36	0.81 – 2.30	0.250
Penalized robust IVW	1.36	0.81 – 2.30	0.250
MR-Egger	1.56	0.49 – 5.02	0.455
Penalized MR-Egger	1.56	0.49 – 5.02	0.455
Robust MR-Egger	1.56	0.82 – 2.94	0.173
Penalized robust MR-Egger	1.56	0.82 – 2.94	0.173

OR: Odds ratio, CI: Confidence interval.
